# Total Face Approach (TFA): A Novel 3D Approach to Describe the Main Cephalometric Craniomaxillofacial Parameters

**DOI:** 10.3390/mps4010015

**Published:** 2021-02-20

**Authors:** Giovanna Perrotti, Giulia Baccaglione, Tommaso Clauser, Luca Testarelli, Massimo Del Fabbro, Tiziano Testori

**Affiliations:** 1 Lake Como Institute, 22100 Como, Italy; giovanna.perrotti@lakecomoinstitute.com; 2 Postgraduate Specialty in Orthodontics, University of Pavia, 27100 Pavia, Italy; giulia.baccaglione@gmail.com; 3 Department of Biomedical, Surgical and Dental Sciences, Università Degli Studi di Milano, 20122 Milan, Italy; tommaso.clauser@unimi.it (T.C.); massimo.delfabbro@unimi.it (M.D.F.); tiziano.testori@unimi.it (T.T.); 4 IRCCS Istituto Ortopedico Galeazzi, 20161 Milan, Italy; 5 Department of Oral and Maxillo-Facial Sciences, “Sapienza” University of Rome, 00161 Rome, Italy; 6 Department of Periodontics and Oral Medicine, University of Michigan, School of Dentistry, Ann Arbor, MI 48109, USA

**Keywords:** 3D cephalometry, vertical size, sagittal dimension

## Abstract

The aim of this study is to propose a 3D skeletal classification and relative normal values of reference. **Method**: from a pool of 271 cone-beam computerized tomography images 108 chin-summit examinations of the skull were selected and divided into 3 traditional skeletal classes. The same Cone-beam Computerized Tomography (CBCT) images were then assessed using the cephalometric multiplanar analysis following the total face approach protocol. **Results**: the results of this study indicate standard 3D cephalometric norms for the vertical and sagittal evaluation of the skull. **Conclusions**: data obtained from our measurements allowed the creation of intervals supplying nosological classification that could be used in orthodontics, orthognatic surgery and implant surgery in fully edentulous patients.

## 1. Introduction

Changing from 2D to 3D cephalometry is not a linear process, it is fraught with obstacles and real problems. Traditional cephalometry is based on linear and angular measurements with uncertain accuracy. It has, nevertheless, supplied clinicians useful information, especially regarding classification. Every author established parameters leading to the internationally recognized skeletal classification of types I, II, and III, but some authors [1] realizing the limits of classifying patients with latero–lateral cephalometry integrated it with a more complex approach adding axial and anterio–posterior crane cephalometry as well. Shifting from 2D to 3D is rendered possible thanks to 3D images obtained employing Cone-beam Computerized Tomography (CBCT).

Swennen and Schurttyser [2] described the advantages and the disadvantages of Multi-slice Spiral Computerized Tomography (MSCT) 3D cephalometry underlining the potential of CBCT 3D cephalometry. Jacobson [3] created a new 3D cephalometric analysis of the anthropometric type both for soft tissues and hard based on a cephalometric system involving three planes of primary reference (Anterior Facial Plane, Lower Facial Plane, Superior Facial Plane).

Gateno [4], with his model of analysis, maintained what is positive in 2D cephalometry but attempted to overcome its limits. In their report, the authors presented a new three-dimenisonal cephalometry to compensate for the unreliability of internal reference systems, and render three-dimensional measurements more accurate, bearing in mind the lack of tools to assess and measure the symmetry. A novel method to evaluate asymmetry was proposed in which size, shape, position, and orientation of the different facial units are measured [5].

De Oliveira et al. [6], Kusnoto [7] Medelnik [8] established that the reproducibility of a landmark differs on the three spatial planes (x; y; z); this means that some points are easily identified on one or two planes but difficult to do so on the ‘z’ plane. The construction of a correct Midsagittal plane has been the focus of various studies. From one study by Damstra et al. [9] it was shown that there are clinically relevant differences between the Midsagittal plane and the real symmetry plane on the images obtained from MSCT and CBCT. Besides, in their study, it was noticed that the Nasion point had a maximum morphometric deviation of less than 0.5 mm from the Midsagittal Plane and may be used as a point of reference for the construction of the plane. Jacobson et al. [3] define the Midsagittal plane as a median plane that divides the head sagittally across the N point when the orientation of the patient, seen frontally, is in a realistic, natural position (NHP). In this case, the Midsagittal plane derived from the true natural position is based on visual perception and does not rely on the internal structures. Examining 2D radiographs of numerous subjects presenting cranio–mandibular asymmetry, Tripkov et al. [10] concluded that the vertical lines constructed as perpendiculars via half points between couples of orbital, bilateral landmarks are more accurate compared to those constructed between two median points. A significant effect has been described on the measurements starting from the Midsagittal plane. It has been shown that the distance between landmarks can influence the degree of error in the measurement obtained and the nearer the two points utilized are the greater tends to be the error in the angular measurement. The great Midsagittal plane variations also in groups of apparently symmetric people can be explained by the fact that perfectly symmetric faces do not really exist, and a certain degree of asymmetry is normal in all human beings. Therefore, median landmarks used in the construction of the Midsagittal planes may deviate from the true plane of symmetry (the one that ideally divides the head into two identical halves on the Sagittal plane). What is more, the combined effect of two or more non-median points due to local remodeling may produce a significant deviation from the true plane of symmetry. This indicates that internal structures even if remodeled may be irrelevant for the visible facial symmetry. The determination of a Midsagittal plane based on median cephalometric points may vary from person to person and remains a controversial method.

The study of facial symmetry is of particular importance in maxillofacial intervention and orthognathics. The fundamental progress from the acquisition of 3D images and visualization via volumetric rendering enables the employment of appropriate cephalometric parameters to measure skeletal structures so as to establish statistical ranges regarding norms and variations. As in traditional 2D cephalometry, there are numerous types of cephalometric analysis with relative norms giving rise to skeletal classifications I, II, and III. In 3D the first step is to establish the most appropriate type of skeletal classifications to carry out 3D measurements [11,12]. It is worth noting that CBCT developed in recent years is also used in cephalometry to study skeletal structures to plan implant-guided surgery [13,14]. The resulting skeletal evaluation could be very useful in assessing the 3D maxillary–mandibular relationship, especially in fully edentulous cases.

The study of vertical and sagittal dimensions and skeletal symmetry/asymmetry values supply detailed information that is not considered in traditional diagnostic protocols and could be useful when planning predictable, complex implant rehabilitation.

The aim of this study is to propose a 3D skeletal classification and relative normal values of reference.

## 2. Materials and Methods

Three equal groups of 36 CBCTs were created after screening the dataset of all the 271 consecutive tomographic exams (prescribed by different specialists and performed by the department of radiology of IRCCS Galeazzi Institute) on the basis of their skeletal classification utilizing the 2D Steiner cephalometric analysis. The subdivision into classes was obtained by tracing angular cephalometric values Sella–Nasion-A point (SNA), Sella–Nasion-B point (SNB) and A point-Nasion-B point (ANB) on latero-lateral teleradiographs reconstruction starting from Digital Imaging and Communications in Medicinefiles (DICOM).

The inclusion criteria included adult subjects (>18 years) with complete dentition or only up to two missing teeth without any occlusal plane alteration. All radiographic exams where the head was not in the Natural Head Position (NHP) [15] were rejected.

Field of view (FOV) was also checked to make sure it was large enough to incorporate all the maxillofacial area from the glabella to menton. The DICOM files utilized in our center come from one CBCT device (NewTom VGI, Verona, Italy) with a voxel size of 150 µm, with a mean adsorbed dose of 100 µSv at full FOV of 15 cm. The DICOM files were uploaded to the Materialise Simplant O&O software (Materialise Co, Leuven, Belgium). The software was equipped with cephalometric analysis capability denoted Total Face Approach (TFA) and was employed to analyze all the cases enrolled in this study.

The 2D cephalometry was carried out on all the selected subjects. The division into skeletal classes was made by tracing the cephalometric exam as outlined by Steiner on sagittal images starting from DICOM of the sample group.

The 3Dcephalometry method employed in this study is of the multi-planar type and requires linear measurements. Three planes of reference were constructed: Axial, Sagittal and Coronal. They are independent of the head posture with which the tomographic exam was carried out.

Every point in these planes is external to the cranium avoiding problems related to local bone remodeling.

A series of construction planes were realized by tracing cephalometric points and made orthogonal and/or parallel among them and with the three planes of reference. The analysis involves a series of linear measurements obtained by calculating the distance between the cephalometric points and constructed planes on the same plane as the three planes of reference of the 3D system: axial, sagittal and coronal planes. The equation used to determine the distance separating any point,

$$d\left(\pi ,\;P\right)=\frac{\left|{\mathrm{ax}}_{0}+{\;\mathrm{by}}_{0}+{\mathrm{cz}}_{0}+d\right|}{\sqrt{a^{2}+{\;b}^{2}+c^{2}}}$$

where a, b, c, d are cephalometric landmarks. For each point, this relationship holds between a cephalometric point and its plane.

The DICOM files used in the research protocol were subdivided on the basis of a skeletal class under examination. The method was divided into two modules.

### 2.1. Vertical Ceph

The vertical CEPH was calculated by the distance between three planes of construction, tracing one cephalometric point parallel to the axial plane. The construction planes are:

- SFP (Superior Facial Plane): plane tracing the Nasio point and running parallel to the axial plane.
- AnSpPl (Anterior Nasal Spine Plane): plane tracing the Anterior Nasal Spine Plane (AnSpl) and parallel to the axial plane.
- MePl (Mental Plane): plane tracing the Menton (Me) point, parallel to the axial plane.

**Figure 1:**
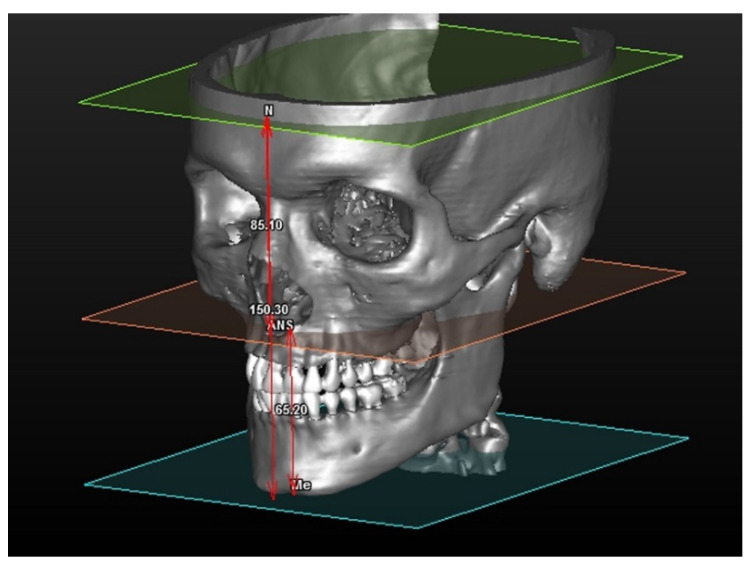
Once planes are drawn, it is possible to measure: (1) Antero-superior vertical dimension (the distance between SFP and anterior nasal spine.), (2) Antero-inferior vertical dimension (the distance between AnSpPl and Mento point) and (3) Anterior total vertical dimension (the distance between the MePl and Nasion point).

The distances are calculated between one point and one plane and are shown in detain in Figure 1.

### 2.2. Sagittal Ceph

**Figure 2:**
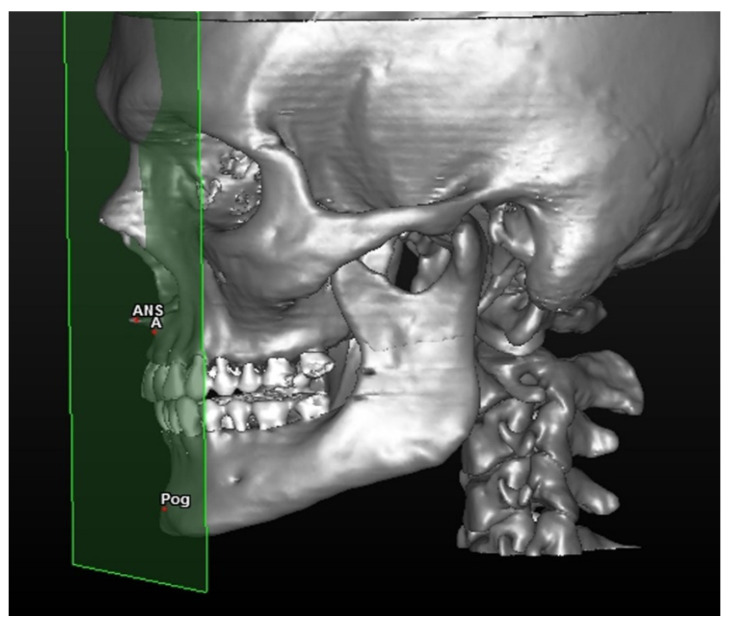
AFP or anterior facial plane traces anterior nasal spine and is parallel to the axial plane. The cephalometric points involved in the analysis are Point A (A) and Pogonion (Po).

The distance between the facial anterior construction facial plane and two cephalometric points (Figure 2).

The linear distance between points A and AFP (defined MX value) and the Pogonion point with AFP (defined MB value) is calculated.

The intermaxillary ratio (IR) is calculated as a difference between the measurements of A–AFP and Pog–AFP.

## 3. Statistical Analysis

Measurements were obtained automatically via the software and saved as.CSV files. They were then exported and copied in a customized Excel table. For each type of measurement of the two modules of the 3D method, a standard deviation was calculated.

The study was conducted retrospectively, utilizing the data-set of the Radiology Department of IRCSS Orthopedic Institute Galeazzi, Milan, analyzing CBCTs already prescribed by the department of orthodontics.

Nevertheless, the study was approved by the Institutional Review Board of the IRCSS Orthopedic Institute Galeazzi as part of a larger research project with exemption from ethical approval, and registered with the Prot. No. 75/2019 (Project Code: L2057).

## 4. Results

In this module 108 patients were analyzed, the three values in the table respectively refer to:

1. Antero-superior vertical dimension (distance between the N and ANS planes)
2. Antero-inferior vertical dimension (distance between the ANS point and Me planes)
3. Total vertical dimension (distance between the N point and Me planes)

**Figure 3:**
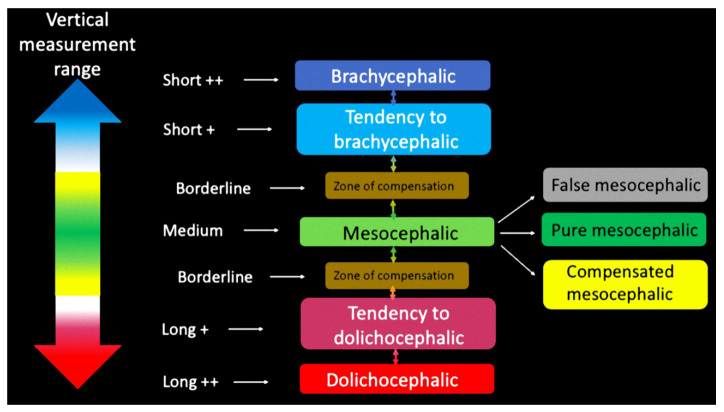
Diagram showing intervals of values associated with a given color which, in turn, indicates a particular diagnosis.

For each of the three values, both the mean and standard deviation were calculated (Table 1, Table 2 and Table 3) and starting from these ranges, six intervals were created. These intervals allowed the subdivision in classes for every single measurement (Figure 3).

- medium: mean ± half of a standard deviation
- Borderline: “medium” ± half of a standard deviation
- Short+/Long+: “borderline” ± a standard deviation.
- Short++/Long++: Short+/Long+ ± a standard deviation.

### Sagittal Dimensions

Regarding the 3D cephalometric method to investigate the antero-posterior position of maxillary base values respectively correspond to:

1. Inter-maxillary ratio (IR): the distance difference between points A and Pogonion (pog) from the anterior facial plane (AFP).
2. Maxilla (MX): measures the distance from point A and to Anterior Facial Plane (AFP)
3. Mandible (MB): measures the distance from Pogonion (Pog) to Anterior Facial Plane

Sagittal dimensions: mean results of sagittal measurements subdivided for skeletal classes I, II and III (Table 4, Table 5 and Table 6).

## 5. Discussion

In the light of the result analysis of our data, it appears that assessment of cranial proportions carried out using 3D images could open new fields of research and can provide much more detailed information on the morphology of skeletal tissues.

Information obtained from cephalometric examination allows the planning of the best possible orthodontic treatment taking into account that osteotomic displacement must achieve not only functional but also aesthetic results. One of the crucial points in cephalometric analysis is the difficulty to fix limits between what is considered normal and deviation from it. Nearly all methods of analysis refer to an ideal facial model; variability in the construction of the cranium is extremely high and its architectural balance can be obtained via numerous, even infinite, possibilities of adaptation among the parts of which it is composed. However, our contribution has some limitations due to the study design: being an observational study, the reader should be aware that the study is relevant for the nosological classification of the skeletal classes. The results of this study could add another factor (a skeletal class classification) to the multiple factors involved in choosing the best treatment plan for each individual patient.

The main drawbacks are the retrospective allocation of patients to the study groups and the small sample size.

The term ‘normality’ is ambiguous as it implies both a concept of ‘ideal’ which varies as our knowledge, aesthetic standards and treatment methods change, and good health, as well as undergoing a certain degree of statistical variation. It is used in one sense or another because it is universally acceptable. It is, however, important not to confuse ‘ideal’ with ‘idol’ which is very common because dictionaries associate ‘ideal’ with ‘imaginary’, contrasting it with ‘realistic’. Orthodontists must therefore be aware that, for most people, a good functional occlusion and pleasant aesthetics are the goal but must not forget that their patient is a unique, sometimes a particular individual, and should be treated as such. These conditions, for most people, can be called ‘normality’, but normality cannot be imposed on everyone and is not always indispensable to enjoying good health and possessing good aesthetics. As Downs writes: *“We can have a value for a given relationship and a value for another relationship that, considered in isolation, may be acceptable; they may combine in such a way as to compensate the disharmony of each other so that the face type appears normal. It may also happen that two values of the same relationship, though in isolation acceptable, may combine badly giving rise to disharmony i.e., an anomaly.”* The quote conceptually is the idea behind the two analyses that allowed the replacement of the ‘normality’ concept with that of ‘compensation’. The 3D study of the cranium starting from the axial, sagittal and coronal images and the corresponding 3D renderings of the volumes made the diagnostic approach much closer to the study of dry skulls. The added value is an opportunity to assess morphology, measurements, position of the live subject and be able to diagnose anatomical parameters on a virtual model that resembles the real subject totally. The necessity for a cephalometric study and the consequent processing of classification may result academic but we believe for the purpose of an orthognathic or implant-prosthetic treatment plan the opportunity to have a nosological framework may be of remarkable help. The possibility to accurately extrapolate the subtle differences that may present in terms of vertical, sagittal and maxillary dimensions shows enormous genetic variation determining the cranio–facial development. The classification we propose is hence only partially comprehensive but is potentially applicable to any population sample with intrinsic variability depending on the ethnic group under examination.

### 5.1. Vertical Dimensions

**Figure 4:**
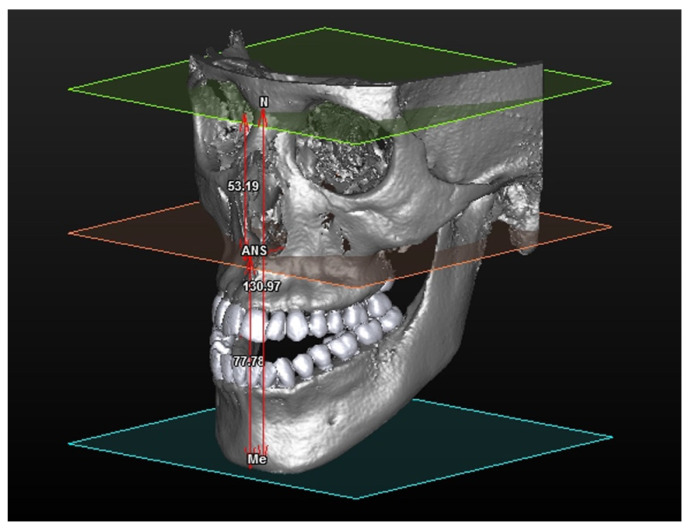
3D rendering of a subject resulting from “Dolicocephalic” analysis.

Processing of data obtained from the three measurements of vertical dimensions allowed the creation of intervals supplying nosological, vertical features of the subject under study (Figure 4).

### 5.2. Sagittal Dimensions

Measurements obtained from statistical analysis of TFA module for antero-posterior cephalometry allowed the nosological subdivision of the sample into skeletal classes in accordance with similar models utilized in traditional 2D cephalometry, but entails some particular differences we introduced (Table 7, Table 8 and Table 9). The Sagittal Ceph and Vertical Ceph are types of analyses that have a profoundly different approach to diagnosis with respect to normal cephalometry methods. This analysis was carried out with a new way of thinking regarding various skeletal structures and their reciprocal relations. This renders it particularly flexible and dynamic; it allows the assessment of various bones not only singularly but also considering the right proportion with others. The 3D view allows the identification of a disharmonious relation among the skeletal bases where a lesser or greater discrepancy occurs in terms of verticality or sagitallity.

## 6. Conclusions

The results of this study could add another factor (a skeletal class classification) to the multiple factors involved in choosing the best treatment plan for each individual patient.

Nevertheless, the TFA approach, with its novel 3D cephalometric analysis allows:

(a) The creation of relative normal values supplying nosological classification.
(b) The assessment of each individual skull bone and their relationships
(c) The 3D reconstruction allows the identification of a disharmonious relation among the skeletal bases.

The three above-mentioned factors could add additional 3D skeletal parameters and the professionals that could benefit from this analysis are:

(1) Orthodontists in their diagnostic phase and in the assessment of the outcomes of the treatment
(2) Maxillofacial surgeons performing orthognathic surgery and assessing its outcome
(3) Implantologist, planning full mouth rehabilitation, especially in totally edentulous patients.

However, the retrospective nature of the allocation of patients to the study groups and the small sample size are biases that should be taken into consideration.

## Figures and Tables

**Table 1:** Results obtained from the analysis of the sample employing the Vertical Ceph 3D method for the superior vertical dimensions (S).

Superior Vertical	Men	Women
Mean	51.65 mm	49.14 mm
Standard Deviation	4.12 mm	2.92 mm

**Table 2:** Results obtained from the analysis of the sample employing the Vertical Ceph 3D method for the inferior vertical dimensions (I).

Superior Vertical	Men	Women
Mean	68.36 mm	61.24 mm
Standard Deviation	6.79 mm	5.46 mm

**Table 3:** Results obtained from the analysis of the sample employing the Vertical Ceph 3D method for the total vertical dimensions (T).

Superior Vertical	Men	Women
Mean	120.01 mm	110.29 mm
Standard Deviation	6.94 mm	6.51 mm

**Table 4:** Results obtained for Skeletal Class I following analysis employing the Sagittal Ceph 3D method.

Skeletal Class I	Sagittal Ceph 3D
RI	−0.24 ± 3.63 mm
MX	4.67 ± 1.43 mm
MB	0.54 ± 9.28 mm

**Table 5:** Results obtained for Skeletal Class I following analysis employing the Sagittal Ceph 3D method.

Skeletal Class II	Sagittal Ceph 3D
IR	−6.05 ± 4.5 mm
MX	4.42 ± 1.72 mm
MB	10.55 ± 4.98 mm

**Table 6:** Results obtained for Skeletal Class I following analysis employing the Sagittal Ceph 3D method.

Skeletal Class III	Sagittal Ceph 3D
IR	6.51 ± 4.62 mm
MX	5.69 ± 1.8 mm
MB	−1.09 ± 5.14mm

**Table 7:** Identification of intervals regarding the spatial evaluation of the upper jaw with respect to the plane coursing the Anterior Nasal Spine (ANS). This spatial value is defined as Maxilla (MX).

MX Value	
Class II	<3.2 mm
Intermediate MX	3.2–4 mm
Mixed	4–6 mm
Class III	>6 mm

**Table 8:** Identification of intervals regarding the spatial evaluation of the mandible with respect to the plane coursing the Anterior Nasal Spine (ANS). This spatial value is defined as Mandible (MB).

MB Value	
Class III	<0.6 mm
Intermediate MB	0.6–3.2 mm
Mean	3.2–5 mm
Class II	>5 mm

**Table 9:** Identification of intervals regarding the reciprocal relation between the upper jaw and mandible. This spatial value is defined as IR.

IR Value	
Class II	<–1.5 mm
Mean	−1.5–2.6 mm
Class III	>2.6 mm

## Data Availability

The data presented in this study are available on request from the corresponding author. The data are not publicly available due to privacy restrictions.
